# Using augmented reality to guide bone conduction device implantation

**DOI:** 10.1038/s41598-023-33523-2

**Published:** 2023-05-03

**Authors:** Justin T. Lui, Valerie Dahm, Joseph M. Chen, Vincent Y. Lin, Jonathan C. Irish, Trung N. Le, Harley H. L. Chan

**Affiliations:** 1 Section of Otolaryngology–Head & Neck Surgery, Department of Surgery, Cumming School of Medicine, University of Calgary, Calgary, Canada; 2 Department of Otolaryngology–Head & Neck Surgery, Temerty School of Medicine, University of Toronto, Toronto, Canada; 3 Guided Therapeutics (GTx) Program, Techna Research Institute, University Health Network, Toronto, Canada; 4 Techna Institute for the Advancement of Technology for Health, University Health Network, 100 College Street, Room 7-207, MaRS Building, Princess Margaret Cancer Research Tower, 7th Floor (STTARR), Toronto, ON M5G 1P5 Canada

**Keywords:** Bone, Surgery

## Abstract

Exact placement of bone conduction implants requires avoidance of critical structures. Existing guidance technologies for intraoperative placement have lacked widespread adoption given accessibility challenges and significant cognitive loading. The purpose of this study is to examine the application of augmented reality (AR) guided surgery on accuracy, duration, and ease on bone conduction implantation. Five surgeons surgically implanted two different types of conduction implants on cadaveric specimens with and without AR projection. Pre- and postoperative computer tomography scans were superimposed to calculate centre-to-centre distances and angular accuracies. Wilcoxon signed-rank testing was used to compare centre-to-centre (C-C) and angular accuracies between the control and experimental arms. Additionally, projection accuracy was derived from the distance between the bony fiducials and the projected fiducials using image guidance coordinates. Both operative time (4.3 ± 1.2 min. vs. 6.6 ± 3.5 min., *p* = 0.030) and centre-to-centre distances surgery (1.9 ± 1.6 mm vs. 9.0 ± 5.3 mm, *p* < 0.001) were significantly less in augmented reality guided surgery. The difference in angular accuracy, however, was not significantly different. The overall average distance between the bony fiducial markings and the AR projected fiducials was 1.7 ± 0.6 mm. With direct intraoperative reference, AR-guided surgery enhances bone conduction implant placement while reduces operative time when compared to conventional surgical planning.

## Introduction

Transcutaneous bone conduction devices (BCDs) are implantable devices that enable acoustic transmission via a magnetic interface with an external sound processor^1–3^. By direct vibratory stimulation of the inner ear, it is widely employed for conductive or mixed hearing loss associated with abnormalities of the middle or external ear in which traditional amplification devices cannot be worn^1–3^. Examples include the BoneBridge floating mass transducer (BB-FMT) made by MED-EL (Innsbruck, Austria) and the Osia osseointegrated steady-state implant (O-OSI) made by Cochlear™ (Sydney, Australia). Although conceptually similar, design and surgical procedures differ.

Exact placement of BCDs requires careful evaluation of the temporal bone contour and thickness. More importantly, critical structures such as the sigmoid sinus or underlying dura should be avoided. Sigmoid sinus injury can lead to substantial bleeding and air embolisms, while dural injury can result in CSF leaks and the development of arteriovenous malformations^3–5^. The use of three-dimensional (3D) planning software allows surgeons to accurately determine ideal BCD positioning while safeguarding against potential injuries and potentially reducing operating times. However, a chasm exists between preoperative planning and surgical performance as manual measurements based off anatomical landmarks introduce room for error^1^.

Several technologies for pre-operative planning and placement of BCDs have been explored. 3D printed transparent stencils interfacing with the surface of the one translated into a mean accuracy near 1.0 mm^3^. However, widespread adoption is lacking possibly due to cost, accessibility, and familiarity with 3D printing. Surgical simulation software paired with intraoperative navigation (IN) tools have also been utilized^2,6^. A major challenge of IN technologies is the omission of relevant information from the surgical field requiring surgeons to switch their focus to and from the surgical field^7^. Augmented reality (AR) guided surgery has been proposed to overcome this by placing relevant information directly into the surgical field^8^.

From guiding bony cuts in oncologic maxillofacial surgery to outlining intradural spinal tumor margins during resection, projected AR technology can help guide surgeons where difficult, atypical anatomy is encountered^9,10^. Given the prospect of underdeveloped anatomy, lack of soft tissue landmarks, and subsurface critical anatomy, AR may be poised to improve BCD surgical performance. We assessed the accuracy of a novel augmented reality (AR) technology to guide placement on cadaveric specimens^10,11^.

## Methods

### Specimen preparation

Whole cadaveric heads were prepared with bilateral curvilinear post-auricular incisions with elevation of a soft tissue flap for exposure of the zygomatic root, posterior external auditory canal, and the mastoid tip. Eight 2 mm bone wells were drilled outside of the surgical field to act as fiducial references for eventual image guidance calibration within the experimental arm. Areas of placement included the zygomatic root, bony external auditory canal, and the mastoid tip.

### Pre-operative planning

Using a prototype intraoperative cone-beam computed tomography scanner (Powermobil, Siemens, Germany), the cadaver heads were obtained, with an isotropic voxel size of 0.78 mm^12^. Scans were evaluated for abnormal anatomy or evidence of previous surgery. Both the O-OSI and BB-FMT devices were imaged for surgical modelling by creating the virtual rendering of hearing device for projecting the overlay during the procedure. Materialise Mimics Medical 19.0 (Materialise NV, Belgium) was used to identify optimal placement of the devices with creation of virtual heads rendered from CT imaging using pre-set bony segmentation sequencing.

**Figure 1:**
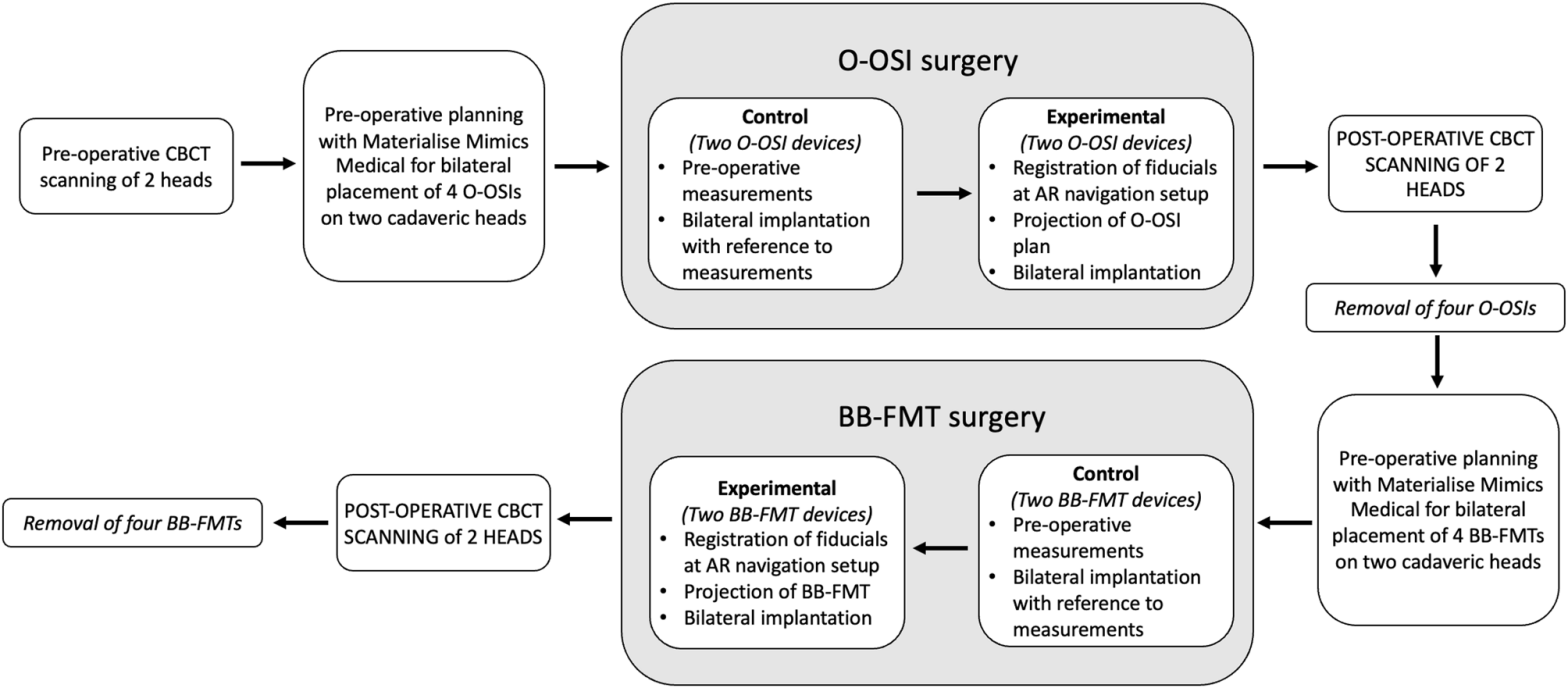
Study protocol *(CBCT* cone beam computed tomography, *O-OSI* Osia osseointegrated implant steady-state implant, *BB-FMT* BoneBridge floating mass transducer).

Implants were imported into Materalise Mimics as optimized triangulated surface meshes that moved independently from the bone. The experimental design is outlined in Fig. 1. Each surgeon’s pre-operative planning included placement of four O-OSI devices and four BB-FMT devices in two separate sessions. Bone depth and avoidance of critical structures, such as the sigmoid sinus were major factors. O-OSIs were placed within the mastoid and clearance around the implant was ensured to avoid inadvertent contact with underlying bone. The three possible placements of the BB-FMTs included the mastoid, retrosigmoid, and middle fossa areas. Each surgeon underwent a brief 10-min session with surgical manuals to review optimal surgical technique for both implants. Each planning session lasted five minutes to allow for surgeons to guide exact placement.

### Bone conduction implantation

**Figure 2:**
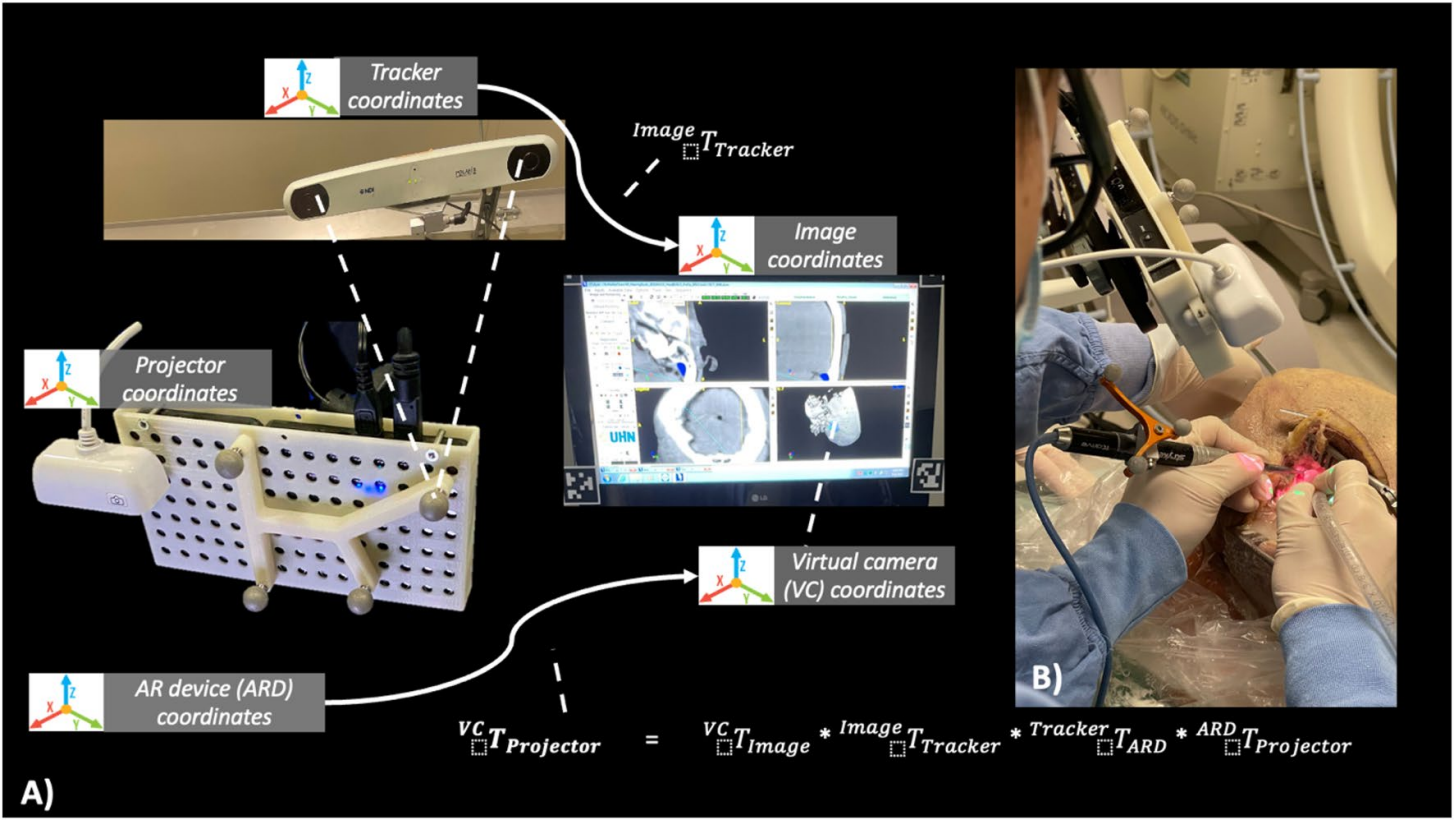
Integrated augmented reality surgical navigation system. (**A**) the projector and surgical instruments were tracked with the optical tracker in reference to the registered fiducials on the cadaveric head. Optical tracking markers attached to the projector allows for real-time adjustments to image projection. The surgical navigation platform displaying a pre-operatively placed implant. Experimental AR projection arm setup. (**B**) Surgeons were encouraged to align the projector to their perspective to reduce parallax.

Implantation followed a standardized protocol beginning with the control arm followed by the experimental AR arm (Fig. 1). Within the control arm, surgeons utilized Materialise Mimics’ built-in measurement tool for eventual intraoperative reference during implant placement. Whereas in the experimental arm, device placement was projected onto the surgical field using GTx-Eyes (Guided Therapeutics, TECHNA Institute, Canada) via a PicoPro projector (Cellon Inc., South Korea)^7,11^. The AR setup is demonstrated in Fig. 2 and seen in the supplementary video.

Following implant placement, CT scans were obtained of the cadaveric heads to capture the location of implantation for eventual 3D coordinates measurement analysis. Each surgeon performed four O-OSI placements followed by four BB-FMTs.

### AR system setup

The integrated AR surgical navigation system consists of a PicoPro projector (Cellon Inc., South Korea), a Polaris Spectra stereoscopic infrared optical tracker (NDI, Canada), a USB 2.0-megapixel camera (ICAN, China), and a standard computer. A 3D printed PicoPro projector enclosure enabled the attachment of four tracking markers, which provide real-time three-dimensional tracking information (Fig. 2). GTx-Eyes (Guided Therapeutics, TECHNA Institute, Canada) is a surgical navigation platform that utilizes open-source, cross-platform libraries included IGSTK, ITK, and VTK^11,13–16^. The developed AR system has demonstrated the projection accuracy at 0.55 ± 0.33 mm and has been widely adapted to the domains of Otolaryngologic and Orthopedic oncologic operations^17–20^. Recently, the software has evolved to include AR integration^7,9^.

The AR system requires two calibrations: (1) camera and instrument tracker, (2) camera and projector, which are both are outlined by Chan et al.^9,11^. The result allows the tracked tool to be linked with the projector’s spatial parameters allowing for both translation and rotational movements.

### Camera and instrument tracker calibration

The camera and tracking tool calibration defines the relationship between the camera’s center and the tracking tool coordinates by creating a homogeneous transformation matrix, ${{}^{Tracker}T}_{Cam}$, consisting of a 3 × 3 rotational matrix **(R)** and a 3 × 1 translational vector **(t)**. The rotational parameter was represented with Euler angles $({R}_{x},{R}_{y},{R}_{z})$. This calibration process requires photographing a known checkerboard pattern from various perspectives using the camera that is affixed to the projector’s case. The instrument tracker’s position and orientation are recorded to compute the spatial transformation. The grid dimensions from each photograph are compared with actual dimensions (30 mm × 30 mm in a 9 × 7 array) using an open-source Matlab camera calibration tool^21^. This calibration serves as the extrinsic parameter of the camera.

The intrinsic parameters **(A)** of the camera include the principal point $({u}_{0,}{v}_{0})$, scale factors ($\alpha , \beta ),\mathrm{ and the skew of the two image axes }\left(c\right)$^22–24^. This is denoted as:

$$\mathbf{A}=\left[\begin{array}{ccc}\alpha & c& {u}_{0}\\ 0& \beta & {v}_{0}\\ 0& 0& 1\end{array}\right]$$

When combining the extrinsic (**R t**) with intrinsic (**A**) parameters, three-dimensional space $({\mathbf{M}=[X,Y,Z,1]}^{T})$ can be mapped to a two-dimensional camera image (${\mathbf{m}=[u,v,1]}^{T}$). *s* is defined as the scale factors. This is represented by: $s\mathbf{m}=\mathbf{A}\left[\mathbf{R} \mathbf{t}\right]\mathbf{M}.$

### Camera and projector calibration

This link defines the spatial relationship between the camera’s centre and the projector to create a homogenous transformation matrix (${{}^{Cam}T}_{Proj}$). A two-dimensional checkerboard image is projected onto a planar checkerboard surface, which was used in the previous calibration step. The camera captures both images from various perspectives. Using the projector-camera calibration toolbox, the transformation of the camera and projector (${{}^{Cam}T}_{Proj}$) is now established^25^. The calibration requires linking the camera and the projector tracking markers, both of which are mounted on the projector enclosure (Fig. 2). By combining both calibration processes, the resulting transformation matrix from the AR projector to the tracking marker is denoted by ${{}^{Tracker}T}_{Proj}={{}^{Tracker}T}_{Cam}*{{}^{Cam}T}_{Proj}$ .

### Projection accuracy

**Figure 3:**
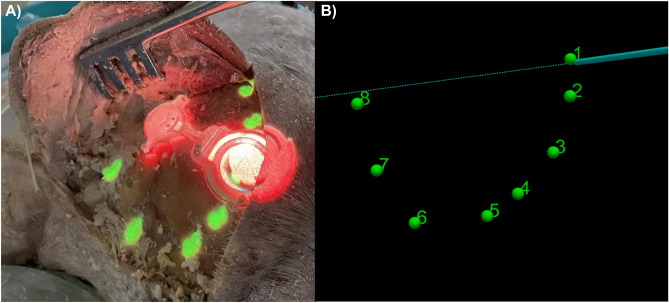
(**A**) Fiducials projection onto the surgical field was matched to the drilled wells and (**B**) subsequent accuracy measurements were obtained with a tracking pointer tool placed within the drilled wells where x-, y-, and z- coordinates were captured.

AR projection setup required confirmation of projection adequacy using an image guidance probe and a Polaris Spectra NDI (Fig. 2). Using the image guidance probe, coordinates from the bony fiducials (drilled bone well) and the projected fiducials (green dots) were captured. The difference between coordinates served as the measurement of projection accuracy (Fig. 3).

### Post-operative measurements

**Figure 4:**
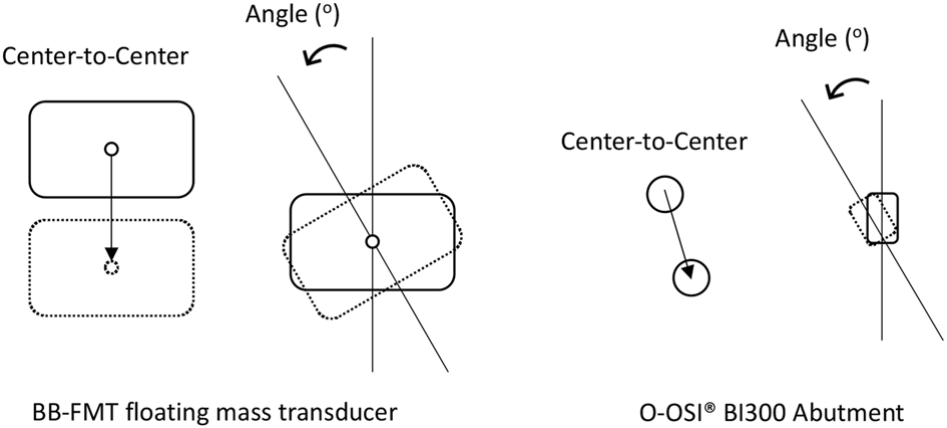
Accuracy measurements for center-to-center distances and angular accuracy.

**Figure 5:**
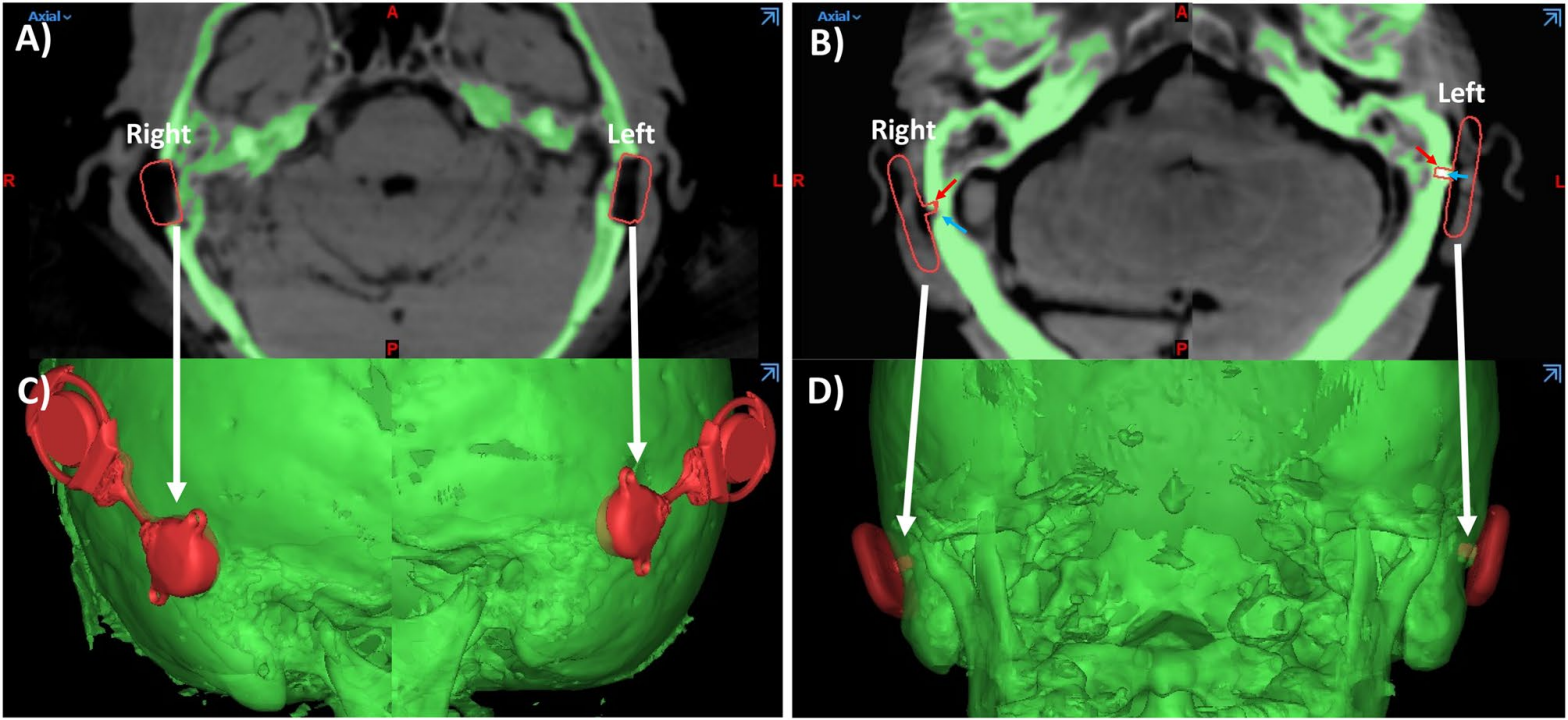
Post-operative CT scans (**A**) BB-FMT and (**B**) O-OSI following AR projector guided surgery with paired pre-operative planning rendering seen in (**C**) and (**D**). In images (**A**) and (**B**), there is the pre-operative planning outline superimposed. The blue arrow denotes post- operative placement whereas the red arrow denotes pre-operative planning.

Post-operative and pre-operative scans were superimposed on Materialise Mimics’ and centre-to-centre distances as well as angular differences on the axial plane were measured (Figs. 4, 5). For O-OSI placements, the centre of the O-OSI was used, whereas the centre of the FMT for BB-FMT.

All participants completed a NASA Task Load Index (TLX) questionnaire assessing the use of AR in addition to providing feedback in an open-ended questionnaire^26^. TLX results were used to generate raw TLX (RTLX) scores for the six domains and subsequently weighted workload scores were generated^27^.

### Statistical analyses

Continuous data was examined for normality by reviewing histograms, quantile–quantile plots, and the Shapiro–Wilk test for normality. Given the lack of normality and repeated measurements, Wilcoxon signed-rank testing was used for centre-to-centre (C-C) and angular accuracies comparisons between the control and experimental arms. All analyses were performed using SPSS 26 (IBM Corp., Armonk, NY).

### Ethics statement

All methods were carried out in accordance with relevant guidelines and regulations. This study was approved by the Sunnybrook Health Sciences Centre Research Ethics Board (*Project Identification Number*: 3541). Informed consent was obtained from all subjects and/or their legal guardian(s) by way of the University of Toronto’s Division of Anatomy–Body Donation Program. All subjects provided consent in the publication of identifying images in an online open-access publication.

## Results

### Surgeons

Three of the five surgeons were fellowship-trained otologists comprising an average of 15 years of experience. The remaining two surgeons were otology fellows. All participants had experience with BB-FMT and O-OSI BI300 placement, respectively.

A Kruskal–Wallis H test was run to determine if there were differences in accuracies between the five surgeons. Distributions of accuracies were not similar for all groups, as assessed by visual inspection of a boxplot. However, the distributions of C-C accuracies lacked statistically significant difference between groups (*p* = 0.857). Similarly, distributions of angle accuracies were not significantly different (*p* = 0.561).

### Fiducial accuracy

The average error in fiducial landmark accuracy within the x-, y-, and z-axes were 0.3 ± 1.1 mm, 0.2 ± 0.8 mm, and 0.6 ± 0.9 mm. This translated to an overall average distance of 1.7 ± 0.6 mm between the bony fiducial markings and the AR projected fiducials.

### Performance

All O-OSI devices were placed in a mastoid position while BB-FMT placement varied with seven FMTs placed in the mastoid, seven in a middle fossa position, and four in a retrosigmoid position. Outlined in Table 1, average operative times were less in the group with AR projection was nearly 2 min faster (4.3 ± 1.3 vs. 6.6 ± 3.5 min., *p* = 0.030). This pattern is consistent when comparing within O-OSI surgeries (*p* < 0.005), but not BB-FMT surgeries (*p* = 0.169).

**Table 1:** Surgical outcomes of both the control and experimental arms (*BB-FMT* BoneBridge floating mass transducer, *O-OSI* Osia osseointegrated steady-state implant).

		Control	AR	Wilcoxon signed-rank test
Operative time (min.)	BB-FMT	6.4 ± 3.7	4.7 ± 1.5	p = 0.169
	O-OSI	6.8 ± 3.5	3.9 ± 0.8	p = 0.005
	Overall	6.6 ± 3.5	4.3 ± 1.2	p = 0.030
Overall center-to-center distance (mm)	BB-FMT	11.1 ± 5.7	1.3 ± 1.7	p = 0.007
	O-OSI	7.0 ± 4.1	2.5 ± 1.2	p = 0.007
	Overall	9.0 ± 5.3	1.9 ± 1.6	p < 0.001
Angular accuracy (°)	BB-FMT	5.1 ± 2.4	3.5 ± 2.4	p = 0.093
	O-OSI	6.8 ± 5.7	6.3 ± 3.1	p = 0.878
	Overall	5.9 ± 4.3°	4.9 ± 3.0°	p = 0.490

Overall C-C distances between the two arms were significantly different (9.0 ± 5.3 mm vs. 1.9 ± 1.6 mm, *p* < 0.001). This trend was seen for both devices with C-C distances consistently less within the experimental arm. For BB-FMT surgeries, the difference between the two arms was 9.8 ± 3.9 mm (*p* < 0.007). For O-OSI surgeries, this difference was less pronounced at 4.5 ± 2.8 mm (*p* = 0.007). In contrast, angular accuracies were not statistically significant between AR and control arms (*p* = 0.490). A significant difference between the two arms when stratifying by device was not evident.

### NASA-TLX and feedback

**Figure 6:**
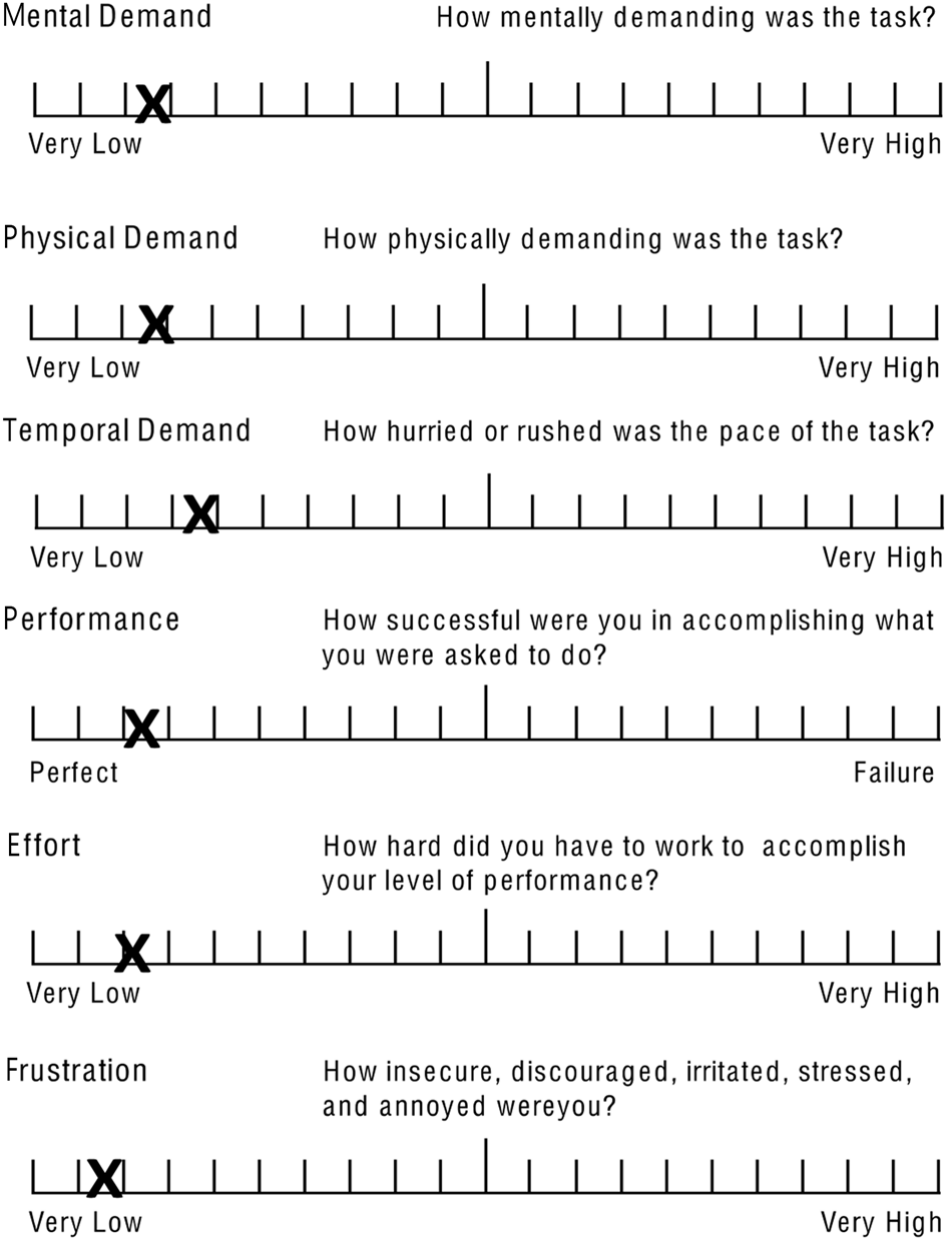
NASA Task Load Index (TLX). Average surgeon raw TLX scores are plotted.

The raw TLX (RLTX) scores for each domain are shown in Fig. 6. Ranked from lowest to highest was frustration (very low: 12), effort (very low: 19), performance (perfect: 21), mental demand (low: 24), physical demand (very low: 25), and temporal demand (low: 33). When factoring in paired-choice weighting scores, the average weighted workload was 19.9. This translates to a “medium” workload when using the AR setup^28^.

Other comments with the technology highlighted the concerns associated with the projector’s light intensity and clarity of the projection borders. Suggestions to overcome blocking of optical tracking markers with bone dust or surgeon placement were to draw projections prior to performing the surgery.

## Discussion

**Figure 7:**
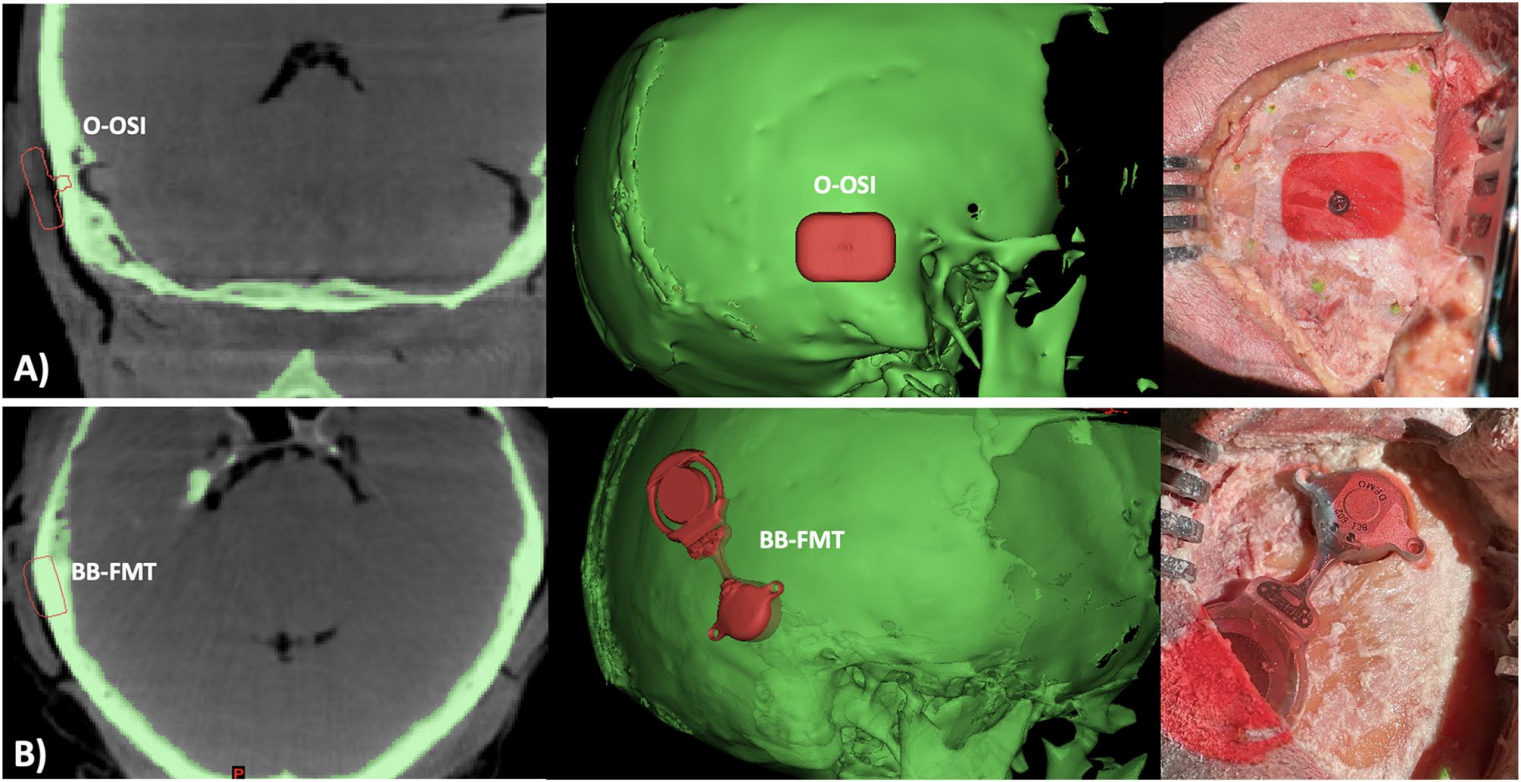
Pre-operative planning of O-OSI and BB-FMT placements. (**A**) Osia osseointegrated implant steady-state implant on coronal imaging with right lateral view. Projection of O-OSI onto the surgical field. (**B**) BoneBridge floating mass transducer on axial imaging with a right oblique view. Projection of the BB-FMT onto the surgical field.

AR guided projection poses a novel technique to bridge the divide between pre-operative planning software and performance of bone conduction implantation surgery (Fig. 7). When comparing C-C distances, AR projection has a clear advantage. The AR system is fully integrated into a surgical navigation platform that utilizes an optically tracked projector and registered fiducial landmarks to avoid bony fixation^11^. The projected image automatically adjusts with any head movement and provides real-time intraoperative reference. Despite being a prototype setup, the AR platform utilizes widely available image-guidance tracking software. Moreover, a central projector prevents the need for wearable technology and allows for multiple viewers from all angles.

Other existing technologies that aim to address pre- and intra-operative planning include 3D printed stencils that provide guidance for intraoperative placement^1,3,5^. Given the ease of sterilization and the added benefit of attaching the guide to the surgical field, these stencils have achieved a high degree of accuracy. Following a successful cadaveric feasibility study, the BB-STAMP (Bone Bridge Surface template-assisted marker positioning) has been performed on three patients with a high degree of C-C accuracy of 0.3 ± 0.1 mm and a rotational accuracy of 4.1 ± 4.7°^5^. A key distinguishing factor, however, was the number of hours dedicated to simulation on physical models and the multi-day process model creation. In contrast, AR projection circumvents the need for a 3D printer or the need for sterilization processing. Moreover, the ability to project critical anatomy such as the sigmoid sinus provides an added layer of patient safety.

For both BB-STAMP and AR projection technologies, rotational accuracy remains a domain requiring continuing improvement. Further iterations of AR projection will include drill trajectory information from optical markers attached to the drill hand pieces as well as a tracking probe that are linked to color projections indicating adequate or inadequate trajectories. Kong et al. described a case study in which CT-guided navigation utilizing an electromagnetic tracker as another means of adapting intraoperative planning for intraoperative reference. However, accuracy and time comparisons were not established^2^.

Other AR technologies have been applied to the head and neck including the use of optical see-through (OST) head-mounted devices (HMD) including the HoloLens™ Gen 1 and 2 (Microsoft Corporation, Redmond, WA)^8^. Advantages of HMD technology over the AR projection includes the convergence of the surgeon’s perspective and field of view without manipulation of the projector or occupying valuable, physical space^29^. Although OST technology preserves the direct view of the world within the HMD, a fixed focal length challenges a user’s ability to maintain focus of both AR and real-world content with more proximal tasks^8,30^. Moreover, performance of precise manual tasks with OST HMD guidance is inferior to performance without AR assistance^30^. The inability to wear surgical loupes or a headlight, battery life concerns, patient privacy, and signal interference have challenged widespread adoption^29^. The use of a projector addresses these shortcomings by enabling multiple viewers from numerous angles and avoiding the need for wearables.

Several challenges exist with AR projection including the need for pre-operative registration to pre-established surface landmarks. In this investigation, 2 mm wells served as bony landmarks for registration. Further research will aim to eliminate this pre-operative preparation with surface registration only. A potential confounder of our results may be attributed to the design of our study order. Specifically, the experimental arm followed the control arm for both implant surgeries. This may have primed surgeons with techniques or nuances that translated to the experimental arm to improve performance.

One of the inherent challenges facing AR projection arises from parallax error^8^. When a surgeon’s perspective and the projection axis are misaligned, the accuracy of surgical implantation accuracy is threatened. The parallax effect, however, was minimized in our AR setup by allowing for surgeons to freely adjust the projector to their viewpoint since the projector’s image would automatically adjust for any movement given its attached location tracker. As a result, recalibration and reregistration were not required following displacement. Given the placement of bone conduction devices along surface anatomy, the parallax has a reduced effect^8^.

The impact of cognitive loading from this novel technology remains consistent across surgeons that AR projection remains low on within the mental, physical, temporal cognitive demands. Both effort and frustration were considered very low with performance considered high. Technological adaptation challenges remain low, enabling surgeons to focus on the surgical task with minimal disruption to their cognitive load.

**Figure 8:**
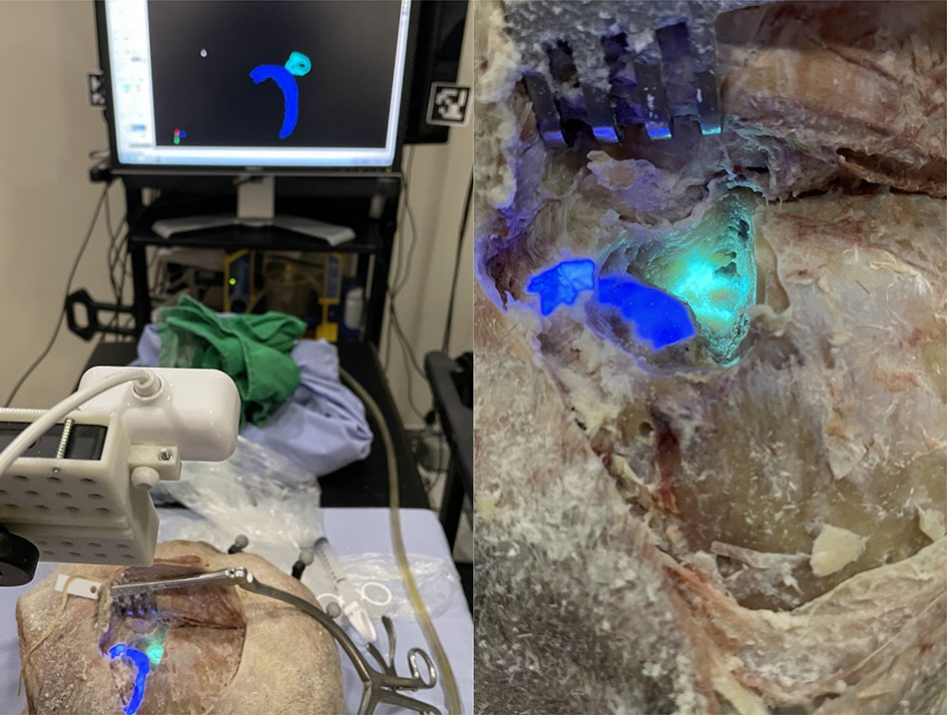
Projection of critical structures encountered in a cortical mastoidectomy including the sigmoid sinus and horizontal semicircular canal.

Future directions include projection of critical anatomy beyond the sigmoid sinus and enable periodic reference of the facial nerve, inner ear, or even the internal auditory canal during other temporal bone procedures (Fig. 8). Additionally, real-time visualization of the insertion trajectory of BCDs and drill tracking may improve angular and placement accuracy, respectively^31^. Future development and research will focus on surface anatomy registration to exclude the need for surface fiducials as well.

## Conclusion

Projection of pre-operative planning onto the surgical field proves to be an exciting opportunity to interact with medical information in a meaningful way. With direct intraoperative reference, AR guided surgery enhances surgical precision when compared to conventional surgical planning while maintaining a low cognitive load on surgeons. In comparison to existing BCD planning technologies such as 3D printed stencils, AR projection does not require time-consuming model production and circumvents the need for sterilization. Its integration into existing surgical navigation platforms while avoiding the challenges associated with head-mounted AR technology reinforces its promise as a useful intraoperative tool.

## Supplementary Information

Supplementary Legends.

Supplementary Video 1.

## Data Availability

The datasets generated during and/or analysed during the current study are available from the corresponding author on reasonable request.
