# Supplementary material for: Using augmented reality to guide bone conduction device implantation
Source: Sci Rep. 2023 May 3;13:7182. doi: 10.1038/s41598-023-33523-2 (PMC10156678; doi:10.1038/s41598-023-33523-2)
Supplement: Supplementary file 1 — Supplementary Legends. [file 41598_2023_33523_MOESM1_ESM.docx]

**Supplementary Video 1.** Augmented reality setup and application in bone conduction implant surgery.
